# A qualitative exploration of stakeholders’ perspectives on the experiences, challenges, and needs of persons with serious mental illness as they consider finding a partner or becoming parent

**DOI:** 10.3389/fpsyt.2022.1066309

**Published:** 2023-01-11

**Authors:** Marine Dubreucq, Paul H. Lysaker, Julien Dubreucq

**Affiliations:** ^1^Centre Référent de Réhabilitation Psychosociale et de Remédiation Cognitive (C3R), Centre Hospitalier Alpes Isère, Grenoble, France; ^2^Fondation FondaMental, Créteil, France; ^3^GCSMS Rehacoor 42, Saint Etienne, France; ^4^INSERM U1290, Research on Healthcare Performance (RESHAPE), Université Claude Bernard Lyon 1, Lyon, France; ^5^Roudebush VA Medical Center, Research Department, Indianapolis, IN, United States; ^6^Department of Clinical Psychiatry, Indiana University School of Medicine, Indianapolis, IN, United States; ^7^Centre de Neurosciences Cognitive, UMR 5229, CNRS, Université Claude Bernard Lyon 1, Lyon, France; ^8^Réseau Handicap Psychique, Grenoble, France; ^9^Service de Psychiatrie de l’Enfant et de l’Adolescent, CHU de Saint Etienne, Saint Etienne, France

**Keywords:** serious mental illness, shared-risk taking, empowerment, recovery, parenting, intimate relationships

## Abstract

**Background:**

While many persons with serious mental illness (SMI) consider intimate relationships and becoming parent as central parts of their lives deeply affecting wellbeing and recovery, others anticipate facing multiple challenges in these life domains. This qualitative study sought to explore the perspectives of persons with SMI and mental health providers (MHPs) with diverse backgrounds and practices on the experiences, challenges, needs and expectations of persons with SMI as they consider finding a partner or becoming parent.

**Methods:**

For this qualitative study, we conducted five focus groups between March and December 2020 for a total number of 22 participants (nine persons with SMI and thirteen MHPs) recruited from a center for psychiatric rehabilitation and a community mental health center in France. We used the inductive six-step process by Braun and Clarke for the thematic analysis.

**Results:**

Participants reported some challenges related to intimate relationships, stigma/self-stigma, disclosure and decision-making about start a family. Their expectations included: (i) psychoeducation about decision-making about finding a partner and starting a family; (ii) support in making empowered decisions about finding a partner, starting a family or disclosure to a prospective partner or their child; (iii) peer-support interventions; (iv) enhancing coping strategies; (v) integrated service provision including home treatment interventions, training to recovery-oriented practices and access to dedicated resources for providers.

**Conclusion:**

In short, intimate relationships and desire to start a family for persons with SMI should be more considered in psychiatric rehabilitation and additional support and interventions should therefore be provided.

## 1. Introduction

Finding a partner and becoming parent are core elements of human experience that are associated with hope, self-esteem, social connectedness, personal recovery and satisfaction with life in people with serious mental illness (SMI) (1). However, while many people with SMI strive to form intimate relationships or to parent, others report challenges related to intimate relationships (2) or to parenting and loss of parenting role.

Research has mostly focused on the barriers to meaningful intimate relationships and the parenting role (e.g., social isolation, stigma and self-stigma, low self-esteem) (1, 2). These barriers might be more pronounced for people with SMI enrolled in psychiatric rehabilitation, where only a minority of persons report to be in relationship or to be parent–one in five men and one in four women in the French REHABase national cohort (3).

While a number of studies have investigated since the late nineties the experiences, views, preferences and support needs of parents with SMI (e.g., two special issues in Frontiers in 2020 and the 3rd edition of a dedicated book) (4, 5), considerably less attention has been paid to their views, expectations and support needs when they consider becoming parent (6). Qualitative research investigated the factors that influence decision-making of women with bipolar disorder about starting a family or illness-related disruption of plans to become a mother in women with SMI (7, 8). However, the perspective of men with SMI on these issues remains largely unknown.

Like many future parents, persons with SMI report concerns regarding their ability to be the parents they wish or wished to be. Compared with those from the general population, future parents with SMI report additional concerns and more non-optimal antenatal caregiving representations [i.e., less expected enjoyment and more fears regarding their caregiving abilities; (8)], which could affect parent-to-infant bonding, the quality of early life parent-infant interactions and finally infant attachment and child outcomes (9, 10). While the peripartum period (i.e., from conception to the child’s 1st year of life) is determinant for parental and child outcomes (e.g., risk for psychiatric, obstetric, and neonatal complications; risk for illness-related interruptions to the parenting role and custody loss) (11), the many parenting interventions developed to support young parents with SMI and their families only focus on what happens after childbirth (12). Recovery-oriented interventions designed to support persons with SMI as they consider becoming parent are still lacking.

Given persons with SMI can see becoming involved in a relationship as a big risk and having a child as an even bigger risk, service provision should include health communication approaches promoting self-determination and person-centered care, e.g., shared-decision making or as recently proposed by Zisman-Ilani (13), shared risk-taking, where patient and provider jointly reflect on the inherent risks of this kind of major life-changing decisions (13). However, mental health providers’ (MHPs) often report feelings of discomfort about discussing these topics with persons with SMI (1–14). These feelings could compromise provider initiation of shared decision-making and lead to more paternalistic approaches, e.g., risk management (13, 14). Despite their importance for recovery, these topics remain overlooked in psychiatric rehabilitation (6).

This qualitative study sought to explore the perspectives of persons with SMI and mental health providers (MHPs) with diverse backgrounds and practices on the experiences, challenges, needs and expectations of persons with SMI as they consider finding a partner or becoming parent.

### 2. Materials and methods

The present study used a qualitative design, using focus groups conducted between March and December 2020. The consolidated criteria for reporting qualitative research (COREQ) (15) were used to design study protocol and report results. Persons with SMI were recruited through a center for psychiatric rehabilitation from the REHABase network (Grenoble). Eligible participants were adults (age > 18) diagnosed with schizophrenia spectrum disorder, bipolar disorder or borderline personality disorder (DSM-5 criteria) (16), not currently in functional remission and willing to give informed consent. MHPs were recruited through a center for psychiatric rehabilitation and a public community mental health center. All providers who cared for persons with SMI and were willing to participate were eligible. The relevant Ethical Review Board (CPP-Ile de France I) approved the appraisal protocol on March 10, 2020.

Five focus groups were conducted for a total number of 22 participants [two groups composed of nine persons with SMI (six males and three females) and three groups composed of 13 MPH]. Focus groups are group discussions where the moderator uses a semi-structured group interview to address specific issues and to ensure that the discussion remains on the subject of interest. Apart from this artificial structure, efforts were made to create a group environment as close as possible to a naturally occurring social interaction. In the present study, participants were asked to discuss the issues, challenges and resources encountered by persons with SMI as they consider finding a partner or becoming parent. They were encouraged to formulate their needs regarding the conception of a dedicated service provision. A semi-structured group interview including probes for the moderator to refocus the discussion if necessary was developed (Supplementary Table 1).

**FIGURE 1:**
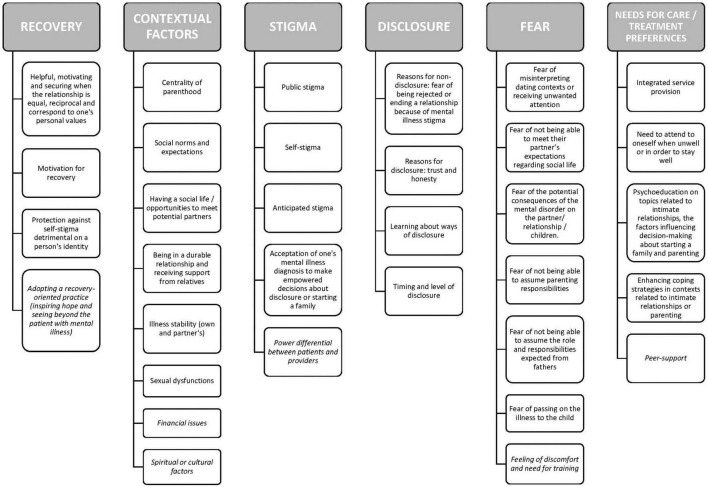
Thematic map of the themes and subthemes [persons with serious mental illness (SMI) and *providers*] about decision-making about intimate relationships and becoming parent.

After participants consented to participate and agreed to the recording of the session, discussions lasted around 2 h. Focus groups were conducted by at least two members of the research team, tape-recorded and fully transcribed. In order to ensure participation of all participants, the 2nd moderator regularly invited those who did not spontaneously contribute to share their experience, thoughts and feelings about the topics covered in the focus group. The first author checked the final transcription against the recordings. For the thematic analysis, we used an inductive, rather than theoretical, approach to qualitatively analyze the data (i.e., “bottom-up” identification of themes) (17). More specifically, we followed the six-step process by Braun and Clarke (17): researchers familiarized themselves with the data as a whole, generated initial codes, searched for themes, reviewed themes, named each defined theme and produced the final report. Themes were refined by re-examining the coherence of data codes within each theme and the validity of each theme in relation with the whole dataset (17). Coder debriefings occurred throughout the analysis to review the identified themes and reach an agreement on coding discrepancies. To allow a deeper and broader understanding of the topic and reduce the risk of interpretation biases, we used investigator triangulation (i.e., independent coding by two researchers, a midwife and a psychiatrist) and data triangulation (i.e., comparison of the perspective of diverse stakeholders on a same topic) (18). However, participants did not give their feedback on the results (reasons not recorded). Code saturation, i.e., the point in the research process where no new information is discovered in data analysis, and meaning saturation, i.e., the point when no further dimensions, nuances, insights of issues can be found (19) were obtained at the end of study. The results of the qualitative analysis are presented on Figure 1 (thematic tree) and Supplementary Tables 2, 3 (quotations supporting the themes and subthemes). The first and the last authors translated the quotes from French, which were then edited by the 2nd author (a native English speaker). Based on the analysis, we developed a recovery-oriented group-based intervention, which is presented on Table 2.

## 3. Results

Five focus groups were conducted (*n* = 22 participants). Most of the nine persons with SMI were men (6, 66.7%) and received a diagnosis of schizophrenia (8, 88.9%). Their mean age was 35.6 (SD = 6.42) years and their mean illness duration of 12.5 years (SD = 6.34). Two were in relationship and parents (22.2%) and one woman was pregnant at the time of the study. Of 13 providers, eight worked at a center for psychiatric rehabilitation (61.5%) and five at a community public mental health center. The provider group was composed of four nurses, four psychologists, two psychiatrists, two social workers (SW 1 and 2), and one professional peer-worker (PW1). Sample characteristics are presented on Table 1. The thematic analysis generated six super ordinate themes: recovery, stigma, disclosure, fear, contextual factors, and needs for care/treatment preferences. The themes, subthemes and illustrative quotes are presented on Supplementary Tables 2, 3.

**TABLE 1:** Sample characteristics.

	People with SMI (*N* = 9)	Mental health providers (*N* = 13)
**Current age**		
Mean (SD)	35.6 (6.42)	35.7 (4.69)
Range	27–45	30–44
Diagnosis*		
Schizophrenia spectrum disorder	8 (88.9%)	–
Bipolar disorder	1 (11.1%)	–
Borderline personality disorder	1 (11.1%)	–
**Illness duration**		
Mean (SD)	12.5 (6.34)	–
Range	7–24	–
**Sex (male)**		
Male	6 (66.7%)	2 (15.4%)
Female	3 (33.3%)	11 (84.6%)
**Education level (years)**		
Mean (SD)	12.4 (2.29)	–
Range	9–17	–
**Marital status (in a couple)**		
No	7 (77.8%)	–
Yes	2 (22.2%)	–
**Parenting status (parent)**		
No	7 (77.8%)	–
Yes	2 (22.2%)	–
**Type of practice**		
Center for psychiatric rehabilitation	–	8 (61.5%)
Community public mental health center	–	5 (38.5%)
**Background**		
Psychiatrist	–	2 (15.4%)
Psychologist	–	4 (30.8%)
Nurse	–	4 (30.8%)
Social worker	–	2 (15.4%)
Peer-worker	–	1 (7.6%)
**Years of experience**		
<3	–	4 (30.8%)
4–6	–	1 (7.6%)
7–9	–	4 (30.8%)
>10	–	4 (30.8%)
**Relatives with SMI**		
No	–	8 (61.5%)
Yes	–	5 (38.5%)

*One participant has two co-occurring conditions (schizophrenia and borderline personality disorder).

**TABLE 2:** Group sessions.

Dating and life in relationship		
**Session number**	**Topic**	**Content**
1	Dating and life in relationship	Introduction: Overview of intervention, exercise asking participant to describe his/her expectations about the intervention and setting group rules Discussing personal representations about dating and life in relationship (e.g., identifying one’s values and expectations about his/her partner, dating and life in relationship; reflecting on what becoming involved in a relationship means for them in their experience of their purpose, position, possibilities, and place in the world) At-home practice exercises
2	Starting and maintaining life in relationship	Introduction: Last session summary/review of last week practices exercises Steps of a relationship Reaching compromises between the partners’ expectations regarding social life and circadian rhythms Explaining one’s needs - including those specific to mental illness - to the partner and taking time for oneself Keeping contact with one’s social network Coping strategies when the relationship is not going well At-home practice exercises
3	Coping with stigma and self-stigma	Introduction: Last session summary/review of last week practices exercises Discussing the impact of stigma and self-stigma in dating contexts/life in relationship Challenging common myths about dating/life in relationship when one partner (or both) have a SMI (e.g., qualities and strengths as a partner and what the person with SMI can bring to the relationship) Story-telling exercises on personal situations or illness-related success stories At-home practice exercises
4	Making empowered decisions about disclosure to his/her partner	Introduction: Last session summary/review of last week practices exercises Pros and cons of disclosure (prospective partner and existing partner) Levels of disclosure (secrecy, selective disclosure, full disclosure; disclosure to the stepfamily/the partner’s friends) Timing and ways of disclosure At-home practice exercises
5	Decoding dating contexts and dating social skills	Introduction: Last session summary/review of last week practices exercises Identifying social cues of interest/disinterest Coping strategies in dating situations Adapting one’s coping strategies to the context (e.g., dating-related vs. SMI-related stress, available resources, dating vs. harassment, coping with rejection) At-home practice exercises
6	Coping with sexual dysfunctions	Introduction: Last session summary/review of last week practices exercises Causes and treatments of sexual dysfunctions (mental and physical health; medications, etc.) Identifying the positive effects of medication (e.g., for social life, life in relationship) Disclosing sexual dysfunctions to one’s partner Discussing sexual dysfunctions resulting from medication and treatment options with his/her physician At-home practice exercises
7	Intimacy-related social skills (I)	Introduction: Last session summary/review of last week practices exercises Attending to a dual set of needs, his/her own and those of the partner Expressing emotions Dealing with anger and social conflicts Coping with break-ups during life in relationship At-home practice exercises
8	Intimacy-related social skills (II)	Introduction: Last session summary/review of last week practices exercises Identifying signs of potential relapses Coping with relapses (e.g., what happens when one or both of the partners have SMI) and the potential consequences of relapse on the relationship (e.g., fear of damaging the relationship; consequences of involuntary hospitalization) Conclusion: summary of group accomplishments, exercise asking participants to describe their feelings following the intervention and to provide feedback on group sessions and content
Optional/ complementary session	What happens next if I choose to not become involved in a relationship?	Introduction: Session overview Identify which factors could contribute to a person’s decision to not become involved in a relationship—either temporarily or definitively (e.g., becoming involved in a relationship could be a source of distress or could be considered as a too big risk to take) Discussing the feelings (e.g., guilt, regret or sense of loss) that may arise from the choice to not become involved in a relationship Taking stock on one’s strengths to redefine a positive sense of identity (e.g., distancing from gendered social norms, roles, expectations and pressure related to the decision to not have children; discussing other facets of a person’s identity, reflecting on how to find purpose and a place in the world without being involved in a relationship, reinvesting other valued social roles) Conclusion: summary of group accomplishments, exercise asking participants to describe their feelings following the intervention and to provide feedback on group sessions and content
**Decision-making about starting a family**		
**Session number**	**Topic**	**Content**
1	What being parent means for me?	Introduction: Overview of intervention, exercise asking participant to describe her expectations about the intervention and setting group rules Discussing personal representations about parenting (e.g., what parenting means for them in their experience of their purpose, position, possibilities, and place in the world) At-home practice exercises
2	Being parent with a SMI	Introduction: Last session summary/review of last week practices exercises Discussing fears about parenting with SMI and potential coping strategies/resources Discussing stigma, self-stigma, common myths about parents with SMI and their impact on a person’s feelings of being legitimate in his/her project to start a family At home practice exercises
3	Coping with stigma	Introduction: Last session summary/review of last week practices exercises Discussing the resources to cope with stigma Story-telling exercises on personal situations or illness-related success stories At-home practice exercises
4	Constructing step by step my project	Introduction: Last session summary/review of last week practices exercises Taking confidence in my abilities to decide whether to start or not a family Discussing the factors influencing the moment when a person would feel “ready” for starting a family Discussing pregnancy or parenting related fears and potential coping strategies or resources At-home practice exercises
5	What about treatment during pregnancy?	Introduction: Last session summary/review of last week practices exercises Making empowered decisions about taking or not a medication during pregnancy Shared-risk taking (i.e., joint reflection on the risks associated to any of the possible decisions) At-home practice exercises
6	Receiving the support I need during pregnancy and after childbirth	Introduction: Last session summary/review of last week practices exercises Identifying stress factors and coping resources during pregnancy and after childbirth Discussing coping resources to reduce stress factors during pregnancy or after childbirth Psychoeducation about symptoms of peripartum depression/alert signs for relapse/coping resources Receiving personalized support adequate to my evolving needs during pregnancy and postpartum At-home practice exercises
7	Taking confidence in my parenting abilities	Introduction: Last session summary/review of last week practices exercises Discussing stress factors related to parenting Identifying the resources (personal, social, environmental and healthcare related) that can support new parents Discussing the dual set of needs related to parenting, a person’s own needs and those of his/her children Opening the child to the outside world At-home practice exercises
8	Making empowered decisions about disclosure to my children	Introduction: Last session summary/review of last week practices exercises Making empowered decisions about disclosure to my children Choosing the best moment to disclose and adapting disclosure to the context and child’s age Conclusion: summary of group accomplishments, exercise asking participants to describe their feelings following the intervention and to provide feedback on group sessions and content
Optional/ complementary session	What happens next if I choose to not have children?	Introduction: Session overview Identify which factors could contribute to a person’s decision to not have children—either temporarily or definitively (e.g., having a child could be a source of distress or could be considered as a too big risk to take) Discussing the feelings (e.g., guilt, regret or sense of loss) that may arise from the choice to not have children Taking stock on one’s strengths to redefine a positive sense of identity (e.g., distancing from gendered social norms, roles, expectations and pressure related to the decision to not have children; discussing other facets of a person’s identity, reflecting on how to find purpose and a place in the world without being parent, exploring the possibilities arising from the decision to not have children and reinvesting other valued social roles) Conclusion: summary of group accomplishments, exercise asking participants to describe their feelings following the intervention and to provide feedback on group sessions and content

### 3.1. Recovery

Most persons with SMI described being involved in an intimate relationship or becoming a parent to be a strong motivation for recovery. These socially valued roles were viewed as factors that could protect against the detrimental effects of self-stigma on their personal identity, contributing thus to wellbeing and personal recovery (i.e., reinvesting life objectives extending beyond mental illness and a devalued and stigmatized view of oneself, known as “illness identity”). Persons with SMI described intimate relationships as helpful, motivating and securing, in particular when the relationship was equal, reciprocal and corresponded to their personal values.

*Female6 (F6): “It’s in how you see yourself, in not reducing yourself to an illness”; Male8 (M8):“it helps a lot to desire to be a parent*… *it is motivating”; M2: “it has to be reciprocal* (…) *mutual help”; F7: “Not first aid* (…*) that’s not a couple anymore, that’s a nurse-patient relationship.”*

Mirroring these findings, most MHPs identified intimate relationship and the desire to have children as key factors that shape a person’s identity. As providers, they considered that their role was: (i) to foster hope by normalizing worries and fears about parenting; (ii) to provide accessible information to help the person make empowered and informed decisions on these topics; (iii) to provide reassurance about a person’s parental abilities to restore a sense of parental identity. Providers reported the need for adopting a recovery-oriented practice to overcome stigma and to see beyond their patients with SMI the partner or the parent the person was or wished to be.

*Peer-worker1 (PW1): “For me, intimate relationships and parenting, it’s also being able to make a free, informed choice* (…*) To choose by oneself, telling: “for me, this will be a good idea, this will be a rewarding experience.”* (…*) To make an informed choice, you need to have accessible information”*; *Social woerker1* (*SW1): “This very much depends on us, on how we perceive a person’s abilities or possibilities* (…*) We see the person as the patient, but we have to investigate the parent she can be or the parent she is.”*

### 3.2. Stigma

Stigma was a major theme running through all the stages of the decision-making process regarding dating, disclosure, forming intimate relationships and starting a family. Several participants were aware of mental illness stigma and anticipated discrimination when dating, deciding to start a family and in assuming the parenting role. Beyond stigma, self-stigma could affect some dating choices and was a barrier to the decision-making process about starting a family or disclosure to the children. Participants discussed the positive and negative aspects of assortative mating (e.g., no fear of being rejected because of their mental illness vs. fear of potential consequences on children if both partners are unwell at the same time).

*F6: “You can tell yourself: ‘I’m mentally ill, it does not even worth thinking about it”’; M9: “Can we be schizophrenic and become parent?”; M9: “How will he understand the illness?* (…*) People with psychiatric illnesses are often apart from others. One way or another, people snigger, you’re sidelined. Normal people don’t want to get along with you. No matter if you have interesting or constructive discussions* (…*) you’re rejected because of the fact “Anyway he’s schizophrenic, all he can say is nonsense”; F7: “My parents*… *I think that they believe I’m not able of taking care for children.” M8: “Do we go on purpose at* (…*) places where you meet other persons suffering from the same illness?”. M2: “That’s why if you live with someone who has the same illness, well, she’ll be more understanding*… *because if she goes through the same difficulties, it’s easier to talk together and to understand each other. Having normal contacts with others it’s hard with the illness”; “F7: If the other one has also a mental illness, which is quite common*, (…*) Knowing how to juggle between the moments you’re unwell and those where that’s him who is unwell. If both are unwell at the same time, it can be, well*…”

Mirroring that, providers identified several barriers to discussing the desire to become parent or the parenting role with their patients such as perceived stigma, experienced stigma and anticipated stigma–including from MHPs -, stigma of seeking help from mental health services and fear of social services involvement but also an asymmetrical relationship between providers and patients at the community center marked by a power differential.

*Psychologist2 (Psycho2): “the fact of not being listened by her doctor who dodges the issue.* (…*) A family who is not really supportive or goes the other way round”; Nurse2: “There is some kind of background*, (…*) well, eugenism, to not discuss that because he would not be able to a good parent”; SW1: “We care for people who live under the medical power for years.”*

Providers described the need for distancing themselves from their own representations/prejudices about their patients’ abilities and skills in their roles as partner or parent in order to be able to provide them a good quality of care. They also reported gender differences in perceived parenting abilities in people with SMI.

*SW1: “we also need sometimes to distance from our representations.* (…*) what does it mean to be a good parent?”; SW1: “Some professionals (from social services) said, “well the person attends to a community mental health center, this means that she’s fragile and can’t get her children back”; Nurse1: “My impression* (…*) is that it’s more complicated when that’s the mother who has psychiatric problems.”*

### 3.3. Disclosure

Disclosure decisions to a prospective partner or to their children were challenging for participants with pros but also cons. Trust and honesty were the main reasons endorsed for disclosure whereas fear of being rejected or ending a relationship because of mental illness stigma were the main reasons endorsed for non-disclosure. Some male participants outlined the need for a person to have accepted his/her SMI diagnosis oneself before disclosing to a prospective partner.

*M5: “it’s better when your partner is aware of the illness*… *She should not be surprised at some weird behavior*… *And if she is ok with it that’s even better”; M8: “Telling her about the periods when I’m not feeling good. She can notice it, and it helps”; M2: “Knowing that I’ll have to tell her that I’m ill often prevents me to approaching a girl”; M5: “you have to make your child understand because he will notice the illness anyway”; M3: “For me you have to accept the illness yourself before the other one can accept it. It makes things more complicated.”*

The timing, level and ways of disclosure were identified as critical issues. Providers also reported this.

*M8: “I told him that when he was a child*… *around 8 years old. And now I think I will go for it and really tell him the word. Schizophrenia”; F7: “when you’ve got a psychiatric illness, you doesn’t necessarily want everyone to know about it”; M5: “Well, you have to be honest with her from the beginning”; M8: “If I say directly “I’m schizophrenic,” I don’t know how the other person will interpret this and if it’s not going well*… *will end the relationship”; Psycho1: “Disclosure: how to tell it, when, to what extent, at which point of the relationship.”*

### 3.4. Fear

Engaging in dating situations was difficult for some participants because of impairments in social cognition and social skills. Some persons feared of misinterpreting social contexts or receiving unwanted attention. Participants reported a dual set of needs, their own and those of their partner or their children. They expressed needing to attend to themselves when unwell or in order to stay well, to care for their children and to be the partner or parent they wished to be.

*M8: “when dating, you have to push yourself forward”; M9: “you have to interpret things accurately* (…*) with the illness, not imagining things that don’t exist”; F6: “I become quite cold and sometimes aggressive because I don’t want to suggest things I don’t feel like”; M2: “Because if you’ve a partner, maybe she would like to go out*… (…*) maybe at one point that will become a source of conflict”; M5: “when you’re ill, sometimes it’s not easy to take on yourself, so taking care of a child.”*

Many of them also worried about the potential consequences of their mental disorder on their partner, on their relationship or on their children. This included the fear of not being able to meet their partner’s expectations regarding social life, fear of not being able to assume parenting responsibilities in case of relapse, fear of being a burden for their partner or child and fears of passing their illness to their child. Some male participants also worried about having difficulties in their fathering role and to set limits to their future children.

*F6: I had several relations that ended because I was too unwell”; F6: “How to cope with distress and delusions*… *How not becoming a burden?; M3: “Well, if you have an illness and the other not*… *you can drag her down too”; M8: “If you’re hospitalized every 3–4 months, the child won’t understand anything anymore”; F6:“it’s turning yourself toward the outside for not being oppressive to the child”; M9: “Do we risk of ruining a child’s life by being ill?”; M2: “And if there are relapses, the person we live with will suddenly have to assume almost alone the baby and our problems”; F4: “My illness is so predominant that I can’t put it aside and take care of my daughter”; M1: “I don’t want my child to live the same things than I and to make him suffer because of me”; M5: “You must not being too strict and you must not be too cool”; M2: “It’s hard to say no* (…*) it’s hard to set boundaries.”*

While providers also identified most of these fears, they also reported feelings of discomfort, loneliness or resourcelessness related to a perceived inability to provide adequate support to their patients with SMI. They expressed concerns about being clumsy when discussing these issues and thus risking damaging the relationship. Some providers described a feeling of additional responsibility, i.e., caring for both the parent and the child. Most providers expressed the needs for improved knowledge, training, and access to an integrated service provision (e.g., information, resources, team work, and a space to share experiences).

*SW1: “This means, as a provider, to feel comfortable enough with these questions”; Psycho2: “I felt resourceless* (…*). So maybe, without being specialist, to know some basics and at least not leaving them blank”; PW1: “I think we’re a bit left alone on that* (…*). I mean, when some people come to us with some serious issues such as custody of children, foster care* (…*) What do we do?”; Psycho1: “in fact they’re looking for reassurance from us and we can’t always reassure them and that is very uncomfortable.”; Nurse3: “There are consequences on the person, meaning that if there are concerns* (…*) that’s something. Not flagging a danger or inappropriately flagging a danger”; Psychiatrist1: “maybe a space for providers* (…*) we don’t have the entire network in mind”; SW1: “Maybe some kind of home treatment team.”*

### 3.5. Contextual factors

Centrality of parenthood was the most important contextual factor influencing the decision to try to become a parent. While becoming parent could be a way to meet social norms and expectations for one participant, others reported that it is not a necessary condition for having a meaningful life and that it should not be the main reason to become parent.

*F7: “the main resource it’s the desire to become parent”; M9 “it’s a stereotype, starting a family, children*… *If you want to step in the society, life means finding someone, have children.”*

Other contextual factors included having a social life and thus more opportunities to meet potential partners, sexual dysfunction resulting from medication, being in a durable relationship and receiving support from family members, friends or providers. Some participants reported needing time for feeling ready to live in relationship or becoming parent.

*M5: “Well, if you conceive a child, it means that the couple is already strong”; M8:“I did talk about it to my physician.* (…*) Well, there are also our parents that can help us with parenting”; M2: “Living in a relationship, for me it’s a major step in life and I personally do not feel ready to cope with it.”*

Another factor influencing the decision to become parent and the choice of the timing was illness stability–their own and those of their partner if he/she also has a diagnosis of mental illness.

*F7: “You shouldn’t have children to cure yourself”; F6: “Well, first to be more stable yourself* (…*) for years and see that you did not had any severe crisis”; F7: “If the other one has also a mental illness, which is quite common*, (…*) Knowing how to juggle between the moments you’re unwell and those where that’s him who is unwell.”*

Providers reported additional contextual factors (e.g., spiritual or cultural factors; financial issues that could result in asymmetrical relationships).

*Psycho1: “taking into account the cultural part, the spiritual part. The context in which evolves the person.”*

### 3.6. Needs for care and treatments preferences

Several participants spontaneously expressed suggestions about the topics that should be addressed in an intervention. They included psychoeducation, e.g., identification of a person’s coping resources as future parent.

*M2: “information about heritability”; F7: “I’m wondering a lot about my ability to be a mother* (…*) actually when you think about becoming parent when you’ve a mental illness, you mostly think about the barriers and not your resources.”*

A pregnant female participant expressed the desire to learn more about potential treatment’s teratogenic effects on her unborn child and expressed concerns about not being adequately supported in case of postpartum depression.

*F7: “Knowing which treatment you can take securely when you’re pregnant.”*

Other suggestions included social skills training on topics related to intimate relationships or parenting, e.g., expressing emotions or dealing with social conflicts.

*M5: “social skills training”; M3: “how to deal with conflicts”; M2: “how to manage anger.”*

Providers identified mostly treatment needs that were already reported by participants with SMI. They included information about pregnancy, childbirth and available resources, communication skills, assertive behaviors, emotion regulation, peer-delivered interventions, psychoeducation and parental guidance.

*Psycho1: “parental guidance”; PW1: “To have answers from a provider if that’s what they’re looking for and at the same time the experience of other parents who were there before”; PW1: “if I take it, who could support me?”; Psychiatrist1: “peer-workers.”*

Some providers also offered coaching for overcoming stigma when communicating with social services–in particular in cases where they perceived the child placement in foster care as inappropriate.

*Psychiatrist1: “Well, her children had been sent to foster care at a moment when they shouldn’t have”; SW1: “you increase the concerns of the professionals, because*… *They start from the point that because you’re seeking care at the community center, you’re fragile. You know it and when you talk to them, you’re freaking out, we don’t understand anything you’re saying. And it harms you because they’re telling themselves “she’s not going well, she’s totally spread out.” So now we stop that* (…*) you make short sentences* (…*). You gave the wrong information”; Nurse1: “she needed to learn how the institution works.”*

### 3.7. Implications for service provision

Based on the qualitative analysis, we developed a manualized, structured, strengths-based 16-session metacognitively oriented (i.e., promoting joint reflection on the information provided in the sessions rather than pure teaching) group-based psychoeducation intervention focused on the decisions to become involved in a relationship or to become parent for people with SMI, conducted by two facilitators. The metacognitively oriented part of the intervention is based on the principles of Metacognitive Reflection and Insight Therapy (e.g., enhancing a person’s ability to form an integrated sense of self and others and to use that sense to respond to the ongoing challenges and possibilities that could arise from personal life experiences) (20). It is composed of two independent 8-session modules, one on intimate relationships and the other on decision-making about starting a family to which participants can attend consecutively or separately depending on their preferences. The program also includes one complementary session per module for those who would decide after the intervention to not become involved in an intimate relationship/to not have children. Table 2 provide an overview of the topics discussed in the sessions.

## 4. Discussion

### 4.1. Main findings

To our knowledge, this qualitative study is one of the first to investigate from the perspective of persons with SMI and MHPs their views, expectations and preferences on the conception of a dedicated service provision to support people with SMI as they consider finding a partner or starting a family.

We found some interaction but also some degree of difference in the identified themes between persons with SMI and MHPs. In particular, although the interview guide addressed similar topics in both groups, persons with SMI (in majority non-parents who never had children) discussed the anticipated challenges of parenting, whereas providers spoke about their experience of caring for parents with SMI. This suggests that for persons with SMI this issue arises earlier than for providers (i.e., before deciding to actually become parent, this contrasting with the perspective of providers where this topic is mostly considered after childbirth), this aligning with the predominance of studies on the experience of parents with SMI in the literature (12, 21). Contrasting with Berger-Merom et al. (14), MHPs did not consider intimate relationships or the desire to start a family as a barrier to shared decision-making.

Both groups reported expectations about a dedicaded service provision for people with SMI as they consider finding a partner or becoming parent that included: (i) metacognitively-oriented psychoeducation and support in making empowered decisions about becoming involved in an intimate relationship or starting a family (e.g., informed decision-making and shared risk taking); (ii) peer-support interventions; (iii) enhancing coping strategies; (iv) support in making empowered decisions about disclosure to a prospective partner or their child. Additional expectations expressed by providers included an integrated service provision including home treatment interventions and access to dedicated resources (e.g., specific information or training to help them answer to the questions of their patients or support in challenging situations).

### 4.2. Interpretation of the findings

In line with previous qualitative research (22, 23), persons with SMI described finding a partner or becoming parent as strong motivating factors that could contribute to wellbeing and recovery. In particular, participants described these topics as central to their experience of their purpose in life, of what is possible for them and of their place in the world. These concepts are related to the construct of metacognition–i.e., the spectrum of activities ranging from discrete mental experiences to the synthesis of intentions, thoughts and feelings in an complex and coherent representation of self and others, which has been recently proposed as a framework for meaning making about life experiences, psychosis-related challenges (e.g., disruptions in plans to become parent) but also for discovering the number of unique possibilities that may spring from these experiences (20).

Extending the results of previous qualitative research (1, 14, 24), MHPs reported feelings of discomfort and needs for additional training when caring for (future) parents with SMI (e.g., fear of being clumsy when discussing these issues with their patients). Of note, MHPs, who agreed on the potential importance of these domains in the process of recovery, outlined the need for adopting a recovery-oriented practice in order to be able to distance from their own representations and to see beyond the patient with SMI the partner or parent the person was or wished to be. This concurs with qualitative research on recovery-oriented practice, i.e., understanding values and treatment preferences and collaborative relationships that support personally defined goals (25). Given many MHPs hold stigmatizing beliefs about their patients’ ability to be involved in an intimate relationship or to start a family–this being a potential barrier to provider initiation of shared decision-making (1, 14), this further supports the need for training them to recovery-oriented practices during the perinatal period (i.e., from preconception to the child’s first year of life). This could imply training to shared risk-taking, where patient and provider jointly reflect on the inherent risks of this kind of major life-changing decisions (13).

We replicated in a predominantly male sample most of the findings of previous qualitative research conducted in female samples on the factors influencing decision-making about starting a family (e.g., degree of social support, anticipated stigma, fear of illness-related consequences on the child or the partner) (1, 7). However, men expressed unique concerns about their ability to fulfill gender-related social expectations related to the fathering role (i.e., dealing with anger when caring for a child and setting limits to a future child), a finding that had not been reported before.

Other gender differences included the timing of concerns regarding the anticipated challenges of becoming parent, i.e., during the perinatal period for women (e.g., the risk for peripartum depression) and during childhood for men (e.g., questions related to education), which likely reflects findings observed in the general population. Of note, MHPs reported more concerns about infant outcomes when the mother had SMI regardless of father’s mental health status. This could concur with gender differences observed in valued social roles that shape a person’s identity (e.g., employment for men–a factor associated to gendered social expectations regarding the fathering role; parenting role for women) (26) and in experienced stigma (e.g., men being perceived as “dangerous” by other people because of their mental illness) (26).

Experienced stigma and/or anticipated stigma related to intimate relationships and parenting abilities–which is common for people with SMI as reported in previous research (27)–contributed to self-stigma that in turn affected decision-making about intimate relationships or about starting a family (e.g., stigma-related non-random mating or disruption in a person’s plan to start a family). Participants discussed the positive and negative aspects of assortative mating and described stigma as a major factor influencing dating choices and opportunities, this aligning with previous research (e.g., anticipated stigma and difficulties to meet people outside of the mental health system) (22). Other negative aspects of assortative mating identified in this study (e.g., fear of potential consequences on children if both partners are unwell at the same time) concur with the literature (e.g., poorer social functioning in partners of persons with psychosis) (28).

Aligning with Ueno and Kamibeppu (29) and Seeman (30), men and women with SMI in this study described challenges in the decision to disclose or not their mental illness to a prospective partner or to a future child. They reported needing support in making empowered decisions about disclosure in these domains (e.g., weighting the pros and cons of disclosure, challenging stigma-related beliefs, levels of disclosure, and deciding the appropriate timing and ways of disclosure), which also concur with Ueno and Kamibeppu (29) and Seeman (30) as well as other studies on decision-making about disclosure in other contexts (31, 32). Peer-led programs supporting people with SMI in their disclosure decisions, e.g., Honest, Open and Proud could be adapted to contexts related to intimate relationships or parenting, although this has–to our knowledge–not yet been realized.

In addition to the treatment needs identified in other studies (e.g., expressing emotions or dealing with social conflicts in intimate relationships (33); peer-support interventions (34); developing strong family and social support networks (1), participants identified the need for an integrative service provision during the perinatal period.

## 5. Limitations

There are limitations. First, while some sample characteristics (age, gender ratio, illness duration) are comparable to larger studies from the REHABase network (3, 6), the nature of the sample recruited could affect the transferability of our findings to people with SMI beyond the study sample (e.g., people with SMI in functional remission not enrolled in the REHABase cohort). Second, although the absence of matching between male and female participants could have reduced the possibility to observe potential gender differences in anticipated challenges and needs, the present study is to our knowledge the first to investigate these issues in a predominantly male sample. Third, while we recruited MHPs from a center for psychiatric rehabilitation and a community MH center, more than one third had lived experience of supporting a relative or a friend with SMI, which might have influenced some findings. However, this proportion is considerably lower than those reported in a national survey of recovery-oriented practice in randomly selected UK community mental health centers (76%) (35). Fourth, while the qualitative analysis of the perspectives of various stakeholders–a method that allows data triangulation and is particularly suitable for an in-depth understanding of a problematic and for designing service provision–is a considerable strength, we couldn’t compare our findings with the experiences of people without SMI. It is therefore possible that some of our findings also apply to people without SMI but also confronted to stigmatization (e.g., people who are HIV-positive) (36). Fifth, although comparing the views of persons with SMI and mental health providers is a strength (code and meaning saturation obtained at the end of the study (19), future studies should explore the perspectives of families, peripartum health providers, childcare health providers and social workers to allow a deeper understanding of these complex issues. Similarly, exploring the experience, ideas and perspectives of other knowledgeable experts on these topics (e.g., marriage counselors, clergy members, matchmakers, and dating site operators) would be of considerable added value. These complementary explorations are scheduled in the near future. Sixth, most of the participants with SMI considering becoming parent never had children, which limits the possibility to extend the present findings to people with SMI who are already parents but consider having another child. Seventh, we did not consider the intersection of multiple forms of stigma (e.g., between mental illness stigma and gender identity stigma), which is another limitation. Eight, most of the persons with SMI already received at least one form of psychosocial treatment at the time of participation. Although none addressed intimate relationship or parenting, this might have influenced some of our findings.

## Data availability statement

The data analyzed in this study is subject to the following licenses/restrictions: The data that support the findings of this study are available on request from the corresponding author. The data are not publicly available due to privacy or ethical restrictions. Requests to access these datasets should be directed to MD, marine.bene@hotmail.fr.

## Author contributions

JD and MD initiated the project, enabled its development and were involved in the collection, and analysis of the data. MD drafted the manuscript. MD and JD the literature review. PL critically revised the article. All authors contributed to and have approved the final manuscript.
