# Supplementary material for: A qualitative exploration of stakeholders’ perspectives on the experiences, challenges, and needs of persons with serious mental illness as they consider finding a partner or becoming parent
Source: Front Psychiatry. 2023 Jan 11;13:1066309. doi: 10.3389/fpsyt.2022.1066309 (PMC9874152; doi:10.3389/fpsyt.2022.1066309)
Supplement: Supplementary file 1 [file Data_Sheet_1.docx]

**Supp. Table 1. Probes used in the semi-structured interview**

**Persons with serious mental illness**

Intimate relationships

What means to become involved in an intimate relationship for you?

Which factors could influence your decision to become involved in a relationship?

Which challenges could you experience as you consider finding an intimate partner?

Which resources could you rely on to face these challenges?

Decision to become parent

What means to become parent for you?

Which factors could influence your decision to become parent?

Which challenges could you experience as you consider becoming parent?

Which resources could you rely on to face these challenges?

What would be your top priorities / your expectations relating the development of a dedicated service provision?

**Providers**

From your experience as provider:

- Which challenges persons with serious mental illness could face as they consider finding an intimate partner or becoming parent?
- What could be their needs for care or additional support in these life domains?

As provider, what could help you in caring for persons with SMI as they consider finding an intimate partner or becoming parent?

What would be your top priorities / your expectations relating the development of a dedicated service provision?
