# Supplementary material for: A qualitative exploration of stakeholders’ perspectives on the experiences, challenges, and needs of persons with serious mental illness as they consider finding a partner or becoming parent
Source: Front Psychiatry. 2023 Jan 11;13:1066309. doi: 10.3389/fpsyt.2022.1066309 (PMC9874152; doi:10.3389/fpsyt.2022.1066309)
Supplement: Supplementary file 2 [file Table_1.DOCX]

**Supplementary table 1. Quotations supporting the themes and subthemes related to recovery, stigma and disclosure**

| **Themes** | **Subthemes** | **Quotations supporting themes and subthemes** |
| --- | --- | --- |
| **Recovery** | **Protection against the detrimental effects of self-stigma** | M9: “it prevents from letting the illness set in”  M5: “despite being ill you can have many qualities and bring her something”  F6: “Trying to see other things and to be more active”  M5: “Either you tell yourself: “well, I’m ill and I’ll stay like that” (…) or you tell yourself “well, I’m ill, but I still want to have a child”… It’s your choice”  F6: “It’s in how you see yourself, in not reducing yourself to an illness” |
|  | **Life in relationship is helpful, motivating and securing.** | M8:“it helps a lot to desire to be a parent… it is motivating”  F6: “It’s a way to cope with life as a couple instead of being alone”  M8: “it is stabilizing to be in a couple… I’m in a couple for 8 years… Listening her voice the morning for instance… it anchors me in reality” |
|  | **Reciprocal relationship corresponding to a person's values and expectations** | F7: “Not first aid… If the other is only here to save you that’s not a couple anymore, that’s a nurse-patient relationship”  M2: “it has to be reciprocal (…) mutual help” |
|  | **Inspiring hope and supporting the development of an identity extending beyond mental illness*** | SW1: “I asked them whether they desired to have children. That their desire to have children was legitimate”.  SW2: “That they can tell themselves that starting a family is not excluded (…) to help the persons to represent themselves a satisfying family life”  Psycho1: “There is a huge need to be reassured on the legitimity of these questions”  Nurse1 “that asks many questions, such as “who am I?” “Am I so different from other people?” (…) Don’t I have the right to a normal life?”  SW1: “It’s always interesting to discuss these questions because it touches something related to the person’s identity (…) it improves the quality of care.”  SW1: “A man who seems to me an adequate parent asks me a lot of questions (…) how at a point he’ll need to be reassured on how he’ll be able to hold his responsibility as parent despite his illness”  SW1: “Our mission is to provide the info the person will need to decide”.  PW1: “For me, intimate relationships and parenting, it’s also being able to make a free, informed choice (…) To choose by oneself, telling: “for me, this will be a good idea, this will be a rewarding experience”. (…) To make an informed choice, you need to have accessible information”  SW1: “This very much depends on us, on how we perceive a person’s abilities or possibilities (…) We see the person as the patient, but we have to investigate the parent she can be or the parent she is”. |
| **Stigma** | **Anticipation to be discriminated when dating, deciding to start a family and in their parenting role because of mental illness stigma** | F6: “You can tell yourself: 'I’m mentally ill, it does not even worth thinking about it'”  F7: “My parents… I think that they believe I’m not able of taking care for children” |
|  | **Self-stigma affects some dating choices and is a barrier to the decision-making process about starting a family or disclosure to the children.** | M2 “To my mind, that’s not compatible”  M8: “when you’re ill and the other not… well she is normal, it’s true too that you put yourself down, asking yourself: "will she accept someone ill?"”  M2: "That’s why if you live with someone who has the same illness, well, she’ll be more understanding… because if she goes through the same difficulties, it’s easier to talk together and to understand each other. Having normal contacts with others it’s hard with the illness”. |
|  | **Public stigma** | F7: “the stigmatizing view of the medias or the society regarding sexuality and mental illness… that being schizophrenic or having a mental illness was being sexually deviant”  M9: “How will he understand the illness? (…) People with psychiatric illnesses are often apart from others. One way or another, people snigger, you’re sidelined. Normal people don’t want to get along with you. No matter if you have interesting or constructive discussions (…) you’re rejected because of the fact “Anyway he’s schizophrenic, all he can say is nonsense”;  F7:“When we talk about people with psychiatric disorders at dinner, it’s often stigmatizing…"  M9:“we’re all painted with the same brush, it is discouraging”  Nurse 2*: “There is some kind of background, maybe what I’m gonna say is a bit too hard, but, well, eugenism, to not discuss that because he would not be able to a good parent (…). Well no, his kids wouldn’t be happy, be this or do that.”  Nurse 2*: “There is a general discourse I sometimes heard saying “well, they can't be parents because they wouldn’t be able to deal with that…” I have the impression that these topics aren’t discussed (…) maybe we should remove the taboo” |
|  | **Worries about being identified as “strange” when dating.** | F7: “My friends already told me that I look too insistently at people’s faces and to stop acting like that because it was destabilizing for others”  M8: “Because I don’t think some will be interested in someone who is too reserved”  M8: “when dating, you have to push yourself forward” |
|  | **Experienced stigma from relatives / providers*** | Psycho1: “her mum clearly dissuaded her from having a 2nd child”  Psycho 2: “the fact of not being listened by her doctor who dodges the issue. (…) A family who is not really supportive or goes the other way round to the fact of living in relationship or having children regardless of the condition. (…) Friends also who can dissuade or who are not willing to discuss that” |
|  | **Asymmetrical relationship between providers and patients at the community center*** | Nurse 1: “people who are under the power of referral to social services (…). There is coercion in mental healthcare (…) the omnipotence of the hospital”  SW1: “We care for people who live under the medical power for years”  Nurse 3: “people involuntarily committed to outpatient care”  Nurse 1 “Something that would be advice (…) and not control. So that there would not be that power.” |
|  | **Need for distancing from one's own representations on intimate relationships or parenting*** | SW1: “As providers we also need sometimes to distance from our representations. (…) When I see the person, I also see who she can be in her social life, her intimate life or in her life as parent. And clearly you always ask yourself: what does it mean to be a good parent?”  SW1: “We can’t reduce the person, because she’s ill to the moment she’s not well. And tell her “well, you’re a bad parent”.”  Nurse2: “There is some kind of background, (…) well, eugenism, to not discuss that because he would not be able to a good parent”  Nurse 1: “Mr X, for example, you can see he’s different. So meeting a girl who is not ill, it was complicated. And there were representations, because I was telling myself “he will only be able to meet a girl who is ill” |
|  | **Gender differences in stigma attached to parents with mental illness*** | Psychiatrist1: “We have many patients who have kids and have severe psychiatric disorders and often that’s rather women who are in charge of daily life (…) the situations I have in mind will more likely be men with children and a woman who can handle (…) who is not ill and deals with daily life”  Nurse1: “Women, well, they often have partners who also have problems.”  Nurse1: “My impression (…) is that it’s more complicated when that’s the mother who has psychiatric problems” |
|  | **Reducing stigma*** | SW1: “This means going outside a place which is labeled “outpatient care”. And providing support to people”  SW1: “Some professionals (from social services) said, “well the person attends to a community mental health center, this means that she’s fragile and can’t get her children back”  SW2: “To raise awareness on that parenting is possible for everyone”  Psycho 2: “before reducing stigma, just raising awareness”  SW2: “we should not again keep people in psychiatry, whereas there is the project to become parent”. |
| **Disclosure** | **Reasons for disclosure: trust and honesty** | M5: “But if you’re already ill and you meet someone… Well, you have to be honest with her from the beginning. And see if she accepts it or not”  M2: “Anyway I think that you must not cheat… It doesn’t worth it, she ‘ll know sooner or later”  M8: “Telling her about the periods when I’m not feeling good. She can notice it, and it helps”  M5: “you have to make your child understand because he will notice the illness anyway” |
|  | **Reasons for non-disclosure: fear of being rejected or ending a relationship because of mental illness stigma** | F7: “it can end the relationship before it actually begins”  F7:“ I’m afraid of his family telling him to leave me, that I’m not good for him”  M9: “How will he understand the illness? How will he understand you’re ill? ”  M8: “how the child will react? Will he reject us too after a while? How will he understand the illness and that we’re ill? (…) Will he accept or not that we’re ill? It’s true that is a bit scary”  M2: “Knowing that I’ll have to tell her that I’m ill often prevents me to approaching a girl” |
|  | **Need to have accepted his / her mental illness diagnosis oneself before disclosing to a prospective partner / deciding to start a family** | M3: “For me you have to accept the illness yourself before the other one can accept it. It makes things more complicated”  M8: “Well, I thought about the responsibility you have towards the child. As you said concerning heredity or the environment you could create around him. But there is also the acceptation of the illness. To accept it yourself.” |
|  | **Timing (e.g. when starting a relationship or depending on the age of the child) of disclosure** | M8: “If you dare to disclose your illness at the beginning of the relationship, it’s something interesting for what happens next”  M5: “Well, you have to be honest with her from the beginning”  M1: “Mine always knew about before, so I never had problems with that”  M8: “Well, he’s 15 years-old. It’s when I told him that I had problems… I had imagined telling him that I had problems in my head, that it was not always simple in my head…. I told him that when he was a child… around eight-years old. And now I think I will go for and really tell him the word. Schizophrenia” |
|  | **Level (e.g. selective disclosure that does not necessarily include a person’s stepfamily) of disclosure** | F7: “his parents are of a generation where a lunatic is a lunatic (…) schizophrenics are murderers”.  F7: “I don't want him to tell his friends about my life. But at the same time, his friends will end noticing that something is wrong with me… that I’m not ordinary… at one time he will have to explain my psychiatric disorder ”  F7: “when you’ve got a psychiatric illness, you doesn’t necessarily want everyone to know about it” |
|  | **Ways of disclosure (e.g. the words used to disclose and explain mental illness)** | F6: “maybe the fact… that we’re kinder with other ill people (…) can help him integrating that the illness is not necessarily what he hears on TV”  M8: “If I say directly “I’m schizophrenic”, I don’t know how the other person will interpret this and if it’s not going well… will end the relationship”  M8: “That’s really important. Because it will help him when you’ll disclose you’re ill yourself”  M3: “You can’t tell him ‘I’m bipolar” or “I’m schizophrenic”. You’ll just say: “Well, Dad has some health problems… sometimes he can be elsewhere but he’ll take good care of you”  M5: “You adapt to your child, you don’t have to tell him everything, you’ll just say “Well, sometimes Dad is a bit ill (…) you help in understand using childish language ”  M8: “I told him that when he was a child… around eight-years old. And now I think I will go for it and really tell him the word. Schizophrenia  Psycho 2: “She was saying “well, what will I tell to my child (…) he’s 5 years old, how will I explain”  Psycho 3*: “explaining the disorder even to the partner (…) sometimes women with borderline personality disorder and co-occurring substance use say “in fact, each time I try to explain, the couple spins out of control” because they don’t really know how to do it, so they do as they can and that’s very scary, so people leave and the next time, they don’t even know whether they should tell it or not or how”  Psycho 1: “Disclosure: how to tell it, when, to what extent, at which point of the relationship”. |

*Themes evoked by providers only
