# Supplementary material for: A qualitative exploration of stakeholders’ perspectives on the experiences, challenges, and needs of persons with serious mental illness as they consider finding a partner or becoming parent
Source: Front Psychiatry. 2023 Jan 11;13:1066309. doi: 10.3389/fpsyt.2022.1066309 (PMC9874152; doi:10.3389/fpsyt.2022.1066309)
Supplement: Supplementary file 3 [file Table_2.DOCX]

**Supplementary table 2. Quotations supporting the themes and subthemes related to fears, contextual factors and needs for care and treatments preferences**

| **Themes** | **Subthemes** | **Quotations supporting themes and subthemes** |
| --- | --- | --- |
| **Fears** | **Fear of misinterpreting dating contexts or receiving unwanted attention.** | M9: “you have to interpret things accurately (…) with the illness, not imagining things that don’t exist (…) To understand well the situation and to handle it”  M8: “Something that actually doesn’t work while you believe it’s working”  F6: “I become quite cold and sometimes aggressive because I don’t want to suggest things I don’t feel like. I don't want people interpreting other things than what I really wish, so it wastes it all”  F7: “if someone does not call back, we’ll immediately imagine he’s rejecting us”  Psycho1*: “some youngsters who can say (…) that they don’t know how to interact with others and in which place they can meet people (…). And how to analyze feedbacks such as “I have no news from that girl, how does I make sense of it?” |
|  | **Need to attend to a dual set of needs.** | M8: “A child is not a crutch (…) you need to go in the same direction than the child, to go in the good direction.”  F6:“ for not using him [the child] as a crutch” |
|  | **Fear of the potential consequences of their mental disorder on their partner, on their relationship or on their children.** | F6: "I had several relations that ended because I was too unwell. Daily life when one of the partners has a mental illness, it’s like juggling with distress… delusions… and most of all the fear of breaking up. (…) it ended because I was in the hospital and to unwell”  M9: “The fact of being ill… To share stuff that can be heavy or boring for her… For not being a burden for her”  M3: “The illness can be a source of conflict in a relationship (…) The relationship shouldn’t be all about on the illness”  M9: “Do we risk of ruining a child’s life by being ill? Not by making him suffer but by making him live this part of… it’s not a cold… it’s not something mild”  M1: “I don’t want my child to live the same things than I and to make him suffer because of me”  M3: “Well, if you have an illness and the other not… you can drag her down too”  M1: “It’s a stress factor, so it can lead to a relapse. If you relapse and you’ve a child… well… (Sigh). (…) For the child, that’s brutal… Well, you’re afraid to hurt him."  F7: “The illness is in the child’s environment, so maybe we’ll transmit him our distress”  M8: “If you’re hospitalized every 3-4 months, the child won’t understand anything anymore”  M2: “And if there are relapses, the person we live with will suddenly have to assume almost alone the baby and our problems”  Psycho 4*: “I can hear sometimes “how could I express my needs to my partner? (…) How can I raise my child without shouting” |
|  | **Fear of not being able to meet their partner’s expectations regarding social life** | F6: “How to cope with distress and delusions… How not becoming a burden?  M2: “Because if you’ve a partner, maybe he would like to go out… And then, you have to be able to cope with going to cinema, to face the crowds. (…) If I prefer sit down on my sofa watching TV, well, maybe at one point that will become a source of conflict and then, well, you have to accept it”  F6: “There is also the issue of tiredness. Because I know I sometimes need to stay at calm and maybe that my partner will want to go out… we will not have the same rhythms”  M8: “Seeing people, going out. For instance, I can’t do it frequently with my girlfriend. Well, we meet others, but maybe not on evenings…. Because I can be a bit tired or mentally unwell.” |
|  | **Fear of not being able to assume parenting responsibilities** | F7: “will we manage to raise our children without giving to much space to our illness in their environment… will we be able to care for children?”  F6:“it’s turning yourself towards the outside for not being oppressive to the child”  M9: “Well, being parent without being ill is already not easy so… You can’t let him down. You can’t just bring him into the world… You can’t afford giving him a disability."  F4: “My illness is so predominant that I can’t put it aside and take care of my daughter”  M5: “when you’re ill, sometimes it’s not easy to take on yourself, so taking care of a child” |
|  | **Fear of not being able to assume the role and responsibilities expected from fathers** | M1: “If I had a child, it would be hard for me to have authority”.  M2: “Sometimes he wants too much to play with me and I don’t want to because I’m an adult and it’s hard to make him understand.”  M5: “You must not being too strict and you must not be too cool”;  M2: “It’s hard to say no (…) it’s hard to set boundaries.”  Psychiatrist 1: “I have sometimes some fathers who bring to the interview some of the difficulties they have with their children (…) it’s on how to set boundaries (…) well rather on educative issues. Some fathers who are resourceless with sometimes a wife who is resourceless too” |
|  | **Fears of passing their illness to their child.** | M1: “Clearly because having an ill child… I would be sorry for him… Because you know what it is (…) And you’d feel guilty too.”  M9: “Will he be ill too? Won’t we pass it on to him?”  M9: “Can we become parents? I asked about the heredity and I’ve been answered to that there would be 80% risk that my child would become schizophrenic”  M2: “Passing on the illness to a child is scary… (…) Regarding my degree of illness, personally I’m afraid”  F6: “And will the child not be himself… Well, will have a mental illness too? Is it something…that is partly genetic?”  Psychiatrist 2: “If I have a mental illness, what are the risks for my child to have it too?” |
|  | **Feelings of discomfort when supporting parents or future parents with mental illness (loneliness, feeling of not being able to provide adequate support, discomfort when discussing the topic)*** | Psychiatrist 1: “these are questions we don’t systematically ask (…) that we’ll discuss because someone will bring it”  SW1: “This means, as a provider, to feel comfortable enough with these questions”  Psycho 2: “I felt resourceless at this moment because I don’t know the numbers and in fact that’s not enough to tell a number. But sometimes the question is so burning, that’s an information they so much desire to have, that they don’t dare to ask, that they ask for the first time and to answer them “well, I don’t know, I can’t answer”… that’s complicated. So maybe, without being specialist, to know some basics and at least not leaving them blank (…) during one month or more and that then, sometimes they won’t dare to ask this question again”  Psycho 1: “I remember this young woman with a diagnosis of schizophrenia (…) who was saying “I wish to be a mum later, can I, can you assure me that my child won’t have anything?” “Well, how to say it? I don’t know”.”  Psycho1: “in fact they’re looking for reassurance from us and we can’t always reassure them and that is very unconfortable (…) Questions are always good but sometimes answers are not necessarily pleasant”  PW1: ““I think we’re a bit left alone on that (…). We are very exposed. And feelings of guilt, I mean, when some people come to us with some serious issues such as custody of children, foster care (…) where does it stop? (…) What do we do?”  Psychiatrist 1: “Well, I think we would need some advice. I know I felt lonely when monitoring pregnancies”  Psycho 1: “Sometimes, the distress they feel is more difficult to handle. (….) Sometimes you feel alone in these interactions.”  Nurse 4: “Not to feel totally resourceless” |
|  | **Feelings of additional responsibility (caring for both the parent and the child)*** | Nurse 3: “There are consequences on the person, meaning that if there are concerns that are raised or dangerousness to the child, the measures that are put in place can be rather radical. Not flagging a danger or inappropriately flagging a danger.”  Psychiatrist 1: “Two other situations ended with a placement in children’s home care, one had a personality disorder, her partner too, they were supporting each other without a child but with a child it exploded (…) And Mrs Y, well she avoided us during her pregnancy (…) It ended badly because it became complicated after childbirth. (…) There has been a referral to social services (…) and an hearing with the judge (…) And my last consultation with her it was “you’re the reason I was convoked to the judge. I doesn’t want to come anymore” (…) I left the door open so that she could came back (…) but at the end we’re feeling powerless”  SW1: “At one point where lies the responsibility? And when it lies on something already problematic, well, not everybody is ready to endorse that (…) the responsibility should be taken before the problem (…). I often have to work on that beforehand, saying “I’m not here to judge you, but to help you and support you, so how do we do to work together so that it works well” |
|  | **Need for specific training on how to support parents or future parents with mental illness*** | Psycho2: “As a provider, skills to avoid, either burning steps or being clumsy. These are things that can be very intimate and if one time we’re clumsy, this can also close things (…) and become far more complicated afterwards, whereas if we are a minimum equipped, we’ll be less clumsy”  Psycho 1: “I need some landmarks. There are questions to which I can’t answer; sometimes they need to have certitudes in contexts where there are none that we can bring  Psycho 1: “To whom and how to refer if we detect something so that it could be more taken into account”  Nurse 4: “To know the network”  Psycho 2: “To share some tools (…) to know what already exists to support that”  PW1: “some notions of law (…) the help of a jurist”  Nurse 2: “There is really a need for more knowledge”  SW2: “Training is central”  Psycho2: “Many providers would be interested in training on that” |
|  | **Need for an integrated service provision supporting parents with mental illness and providers*** | SW2: “that’s why we shouldn’t stay alone”  Psychiatrist 1: “maybe a space for providers, where we could discuss together, where we can bring situations in which we’re feeling stuck or we find borderline. And where whe could discuss with other persons who have different skills, because we don’t have the entire network in mind.”  SW1 “Maybe some kind of home treatment team which would go towards the persons”  Psycho 1 "if we were several to deal with these issues that are so crucial and emotionally charged, this could be less emotionally complicated.  Psychiatrist 1: “I had some complicated discussions with obstetricians who did not always listen or were panicked (…) I found there was a lack of communication” |
| **Contextual factors** | **Centrality of parenthood** | F7: “ this desire that has been present for years”  F7: “the main resource it’s the desire to become parent” |
|  | **Impact of social norms and expectations on decision-making about starting a family** | M2 “Living in relationship, children, all that stuff, it means having a role… in the society… Somehow it’s being normal because it’s the case of most people”  M9 “it’s a stereotype, starting a family, children… If you want to step in the society, life means finding someone, have children and then becoming grand-parent”  F7: “You shouldn’t have children to cure yourself (…) that should not be your main reason to have children” |
|  | **Having a social life and thus more opportunities to meet potential partners** | F6: “I personally do not often go out so I don’t have many occasions”  Nurse 1: “at the end he met a girlfriend at the hospital”. |
|  | **Sexual dysfunction resulting from medication** | M2: “It reduces strongly the libido. At the beginning. I found it hard to accept taking medications because it suppressed everything”  M3: “It has a role and it made difficult for me to take the medication”.  F7: “is it just men who have erectile problems or are we also inhibited in that domain? I don’t know anything about it”  Psychiatrist 1*: “he stopped his medication because of too much effects on erections (…). I try to be more systematic on treatment side effects on that, but still it’s not always the case (…) sometimes we don't dare to ask” |
|  | **Being in a durable relationship** | F7: “The fact to be in an equilibrate, strong relationship with the person with whom you’ll have a child”  M5: “Well, if you conceive a child, it means that the couple is already strong” |
|  | **Feeling ready to live in relationship or becoming parent.** | F6: “In fact, because it’s a big responsibility, you should try to confront yourself step by step to responsibilities (…) and finally you’ll manage to tell yourself ‘yes, I can assume the responsibility to be a parent”  M2: “Living in a relationship, for me it’s an major step in life and I personally do not feel ready to cope with it” |
|  | **Receiving support from relatives or providers.** | M8: “I did talk about it to my physician. (…) He can guide us a bit”  M8:“Well, there are also our parents that can help us with parenting” |
|  | **Illness stability - their own and those of their partner if he /she also has a diagnosis of mental illness.** | F7: “If both are unwell at the same time, it can be, well…”  M3: “For me, you have to make a choice and tell yourself: “should I wait to get better before having a child or should I do it anyway?”  F6: “Well, first to be more stable yourself (…) for years and see that you did not had any severe crisis”  M5: “It depends on the impact of the illness… (…) if you don’t manage to get out yourself, then it doesn’t even worth to think about becoming parent. While you can better take care of yourself when the illness is stable”.  F7: “If the other one has also a mental illness, which is quite common, (…) Knowing how to juggle between the moments you’re unwell and those where that’s him who is unwell”. |
|  | **Spiritual or cultural factors*** | Psycho 1: “taking into account the cultural part, the spiritual part. The context in which evolves the person”  Psycho 2: “the relationship between affective life, parenting and spirituality. Sometimes it’s not the same understanding and again we can be very clumsy”. |
|  | **Asymmetrical relationships related to financial issues*** | PW1: “For me what blocks is (…) the possibility to be financially independent”  SW2: “when someone has a disability allowance and only one of the partners work, that’s complicated to imagine a family life (…) we make people dependent from their partners” |
| **Needs for care and treatments preferences** | **Psychoeducation in general and on specific topics (identification of a person’s coping resources as future parent, heritability and treatments’ side effects on sexuality or fertility and potential teratogenic effects and available support during pregnancy).** | M2: “Information on relapses (…) to notice your symptoms and be able to discuss it with your physician to readapt the treatment”  M3: “And information about that and the consequences of the illness on daily life in relationship”  F7: “Would we be basically able to conceive a child? The treatments we’re taking for years, do they reduce the fertility?”  F7: “Knowing which treatment you can take securely when you’re pregnant”  F7: “Would I be supported in case I get depressed during or after pregnancy?”  M2: “information about heritability”  F7: “I’m wondering a lot about my ability to be a mother (…) actually when you think about becoming parent when you’ve a mental illness, you mostly think about the barriers and not your resources”  PW1*: “Inform on (…) the disorder (…) the treatments and their consequences (…). Inform on a person’s rights and on the available resources, meaning those that could be of help.”  PW1: “if I take it, who could support me?”  Nurse 1*: “Women’s questions, pregnancy, does childbirth hurts?”  SW1*: “What is happening? I have something in the belly”, well that’s normal”  Psychiatrist 2*: “Am I able to? Am I willing to take that risk or not?”  Psycho2*: “she says “well, how will I do if I have a 2^nd^ child? She ended as a single mother and was saying clearly that (…) she did not receive support for the first one (…) she says “how could I have help to anticipate the 2nd one if one day I desire to have a 2nd one?” And she did not know how to do (…) she needed resources beforehand and to anticipate the relationships with the brother or sister to come”  Psycho1: “parental guidance” |
|  | **Family psychoeducation*** | PW1: “a tool that would allow the relatives to access to this information” |
|  | **Peer-support*** | PW1: “it could be very interesting to have testimonies for service users (…) who came to talk about their fathering or mothering role”  Nurse3: “support groups for future parents”  PW1: “To have answers from a provider if that’s what they’re looking for and at the same time the experience of other parents who were there before”  Psychiatrist1: “peer-workers”. |
|  | **Enhancing coping strategies in contexts related to intimate relationships or parenting, e.g. expressing emotions or dealing with anger and social conflicts.** | M3 “What would be great would be learning about how to deal with conflicts…”  M2 “And as previously said how to manage anger (…) when you’re angry, you should try to have a dialogue (…) To develop communication skills like what we do in social skills training”  M5: “social skills training”;  M3: “how to deal with conflicts”;  M1 and M3: “To cope with conflicts”  M3: “To take confidence, to learn how to tell what you’re feeling inside”  F6: “Theater. I think it could actually help to find means of expression. Because I sometimes have difficulties to speak, to express myself (…) I always wonder about the attitude I should adopt. So maybe practicing theater, having tools to rely on. And then be more natural”  M8: “To practice talking exercises in which you have difficulties”  Psycho 3*: “people who have marital difficulties, in fact that’s everything related to communication in a couple”  Psycho 4*: “the first need (…) that’s really communication skills (…) self-esteem, assertive behaviors, communication, emotion regulation”  Psycho 2*: “she told clearly “I don't know how to manage it, how to interact with my baby, I don’t know what to do”, she withdrew into herself, she did what she could (…) she told “couldn’t I learn some things about social cognition?” to better interact with her 5-year old child who was under the father’s custody. And she worried about anticipating the moment her child would come for the weekend telling “I have to try to thing to everything that is material to be able to focus on my child’s needs when I’ll see him and what I’ll be able to decode”  Psycho 1*: “very much parental guidance (…). I don’t know, psychoeducation, things in the same time pragmatic and more informative that can bring skills for patients, relatives and providers” |
|  | **Coaching for overcoming stigma when communicating with social services*** | Psychiatrist 1: “Well, her children had been sent to foster care at a moment when they shouldn’t have”  SW1: “you increase the concerns of the professionals, because… They start from the point that because you’re seeking care at the community center, you’re fragile. You know it and when you talk to them, you’re freaking out, we don’t understand anything you’re saying. And it harms you because they’re telling themselves “she’s not going well, she’s totally spread out”. So now we stop that (…) you make short sentences (…). You gave the wrong information”  Nurse 1: “she needed to learn how the institution works” |

***Themes evoked by providers only**
